# Multiplex Detection of Infectious Diseases on Microfluidic Platforms

**DOI:** 10.3390/bios13030410

**Published:** 2023-03-21

**Authors:** Fumin Chen, Qinqin Hu, Huimin Li, Yi Xie, Leshan Xiu, Yuqian Zhang, Xiaokui Guo, Kun Yin

**Affiliations:** 1 School of Global Health, Chinese Center for Tropical Diseases Research, Shanghai Jiao Tong University School of Medicine, No. 227 Chongqing South Road, Shanghai 200025, China; 2 One Health Center, Shanghai Jiao Tong University—The University of Edinburgh, Shanghai 200025, China; 3 Department of Surgery, Division of Surgery Research, Mayo Clinic, Rochester, MN 55905, USA; 4 Microbiome Program, Center for Individualized Medicine, Mayo Clinic, Rochester, MN 55905, USA

**Keywords:** microfluidic platforms, multiplex detection, infectious disease diagnosis, immunosensors, nucleic acid sensors

## Abstract

Infectious diseases contribute significantly to the global disease burden. Sensitive and accurate screening methods are some of the most effective means of identifying sources of infection and controlling infectivity. Conventional detecting strategies such as quantitative polymerase chain reaction (qPCR), DNA sequencing, and mass spectrometry typically require bulky equipment and well-trained personnel. Therefore, mass screening of a large population using conventional strategies during pandemic periods often requires additional manpower, resources, and time, which cannot be guaranteed in resource-limited settings. Recently, emerging microfluidic technologies have shown the potential to replace conventional methods in performing point-of-care detection because they are automated, miniaturized, and integrated. By exploiting the spatial separation of detection sites, microfluidic platforms can enable the multiplex detection of infectious diseases to reduce the possibility of misdiagnosis and incomplete diagnosis of infectious diseases with similar symptoms. This review presents the recent advances in microfluidic platforms used for multiplex detection of infectious diseases, including microfluidic immunosensors and microfluidic nucleic acid sensors. As representative microfluidic platforms, lateral flow immunoassay (LFIA) platforms, polymer-based chips, paper-based devices, and droplet-based devices will be discussed in detail. In addition, the current challenges, commercialization, and prospects are proposed to promote the application of microfluidic platforms in infectious disease detection.

## 1. Introduction

Infectious diseases are caused by pathogens, including viruses, bacteria, and parasites [1]. As one of the greatest threats to human health and global security, infectious diseases contribute the most to the global disease burden [2,3]. For example, according to the World Health Organization, severe acute respiratory syndrome coronavirus 2 (SARS-CoV-2) had infected 6 billion people and caused 6 million deaths by December, 2022 [4]. Compared to other diseases, infectious diseases are characterized by their high infectivity [1]. Managing their infectivity is crucial for preventing and controlling the pandemics of infectious diseases [5,6,7]. It is reported that timely diagnosis can help control the source of infection by isolating and treating infected individuals. A rapid, sensitive, and accurate diagnostic assay is urgently required to effectively investigate and locate the source of infection and control the spread of infectious diseases [7].

Laboratory tests usually detect infectious diseases with methods such as enzyme-linked immunosorbent assays (ELISA), DNA sequencing, and qPCR [8]. These techniques usually require well-trained operators, professional procedures, and expensive testing equipment [9]. For example, ELISA technology requires a complex labeling procedure and a bulky optical reader [10,11]. As the other gold standard technology for nucleic acid detection [12], qPCR is limited by its many manual steps and precise equipment such as thermocyclers [13]. Therefore, current laboratory detection technologies are struggling to meet the need for rapid mass screening and surveillance of infectious diseases, especially in resource-limited settings. In addition, in many cases (e.g., to distinguish between the different types of sepsis), clinical evidence based on a single biomarker is insufficient to adequately diagnose a disease or monitor its treatment [14]. Moreover, simultaneous detection of infectious diseases with similar symptoms (e.g., respiratory viruses) can rapidly identify the source of infection [15]. In this context, there is a great need for multiplex detection platforms, that can ensure the performance and quality requirements of diagnostics of infectious diseases that can be performed in a short time by laypersons [14,16].

**Figure 1:**
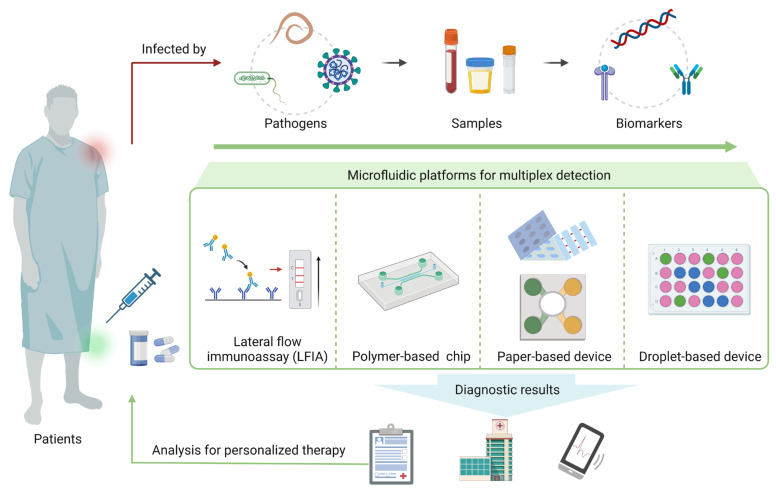
Overview of microfluidic platforms for multiplex detection of pathogens.

Recently, microfluidics has been highlighted and extensively researched due to its outstanding advantages, such as fast operation (less than 1 h), low reagent volume (microliter or even nanoliter), and high integration capability (integration of sample preparation, detection, and analysis into the platform) [17]. In addition, the characteristics of microfluidic platforms are well-suited for multiplex detection [14,18]. The general strategies of multiplex detection include spatial separation of detection sites, discrete regions of a channel network or array, or the detection of different markers in integrated microfluidic chips which can effectively reduce the interference between different reaction systems in the simultaneous detection of a large number of targets [14]. Moreover, because of the relatively independent reaction space on the microfluidic platforms, the sensitivity and specificity of each assay can be ensured in multiplex detection. Therefore, multiplex detection technology based on microfluidic platforms shows great potential for the diagnosis and mass screening of infectious diseases [19,20]. Although there are several reviews that comprehensively discuss microfluidic detection technologies for infectious disease diagnosis [21,22,23], few of them focus on microfluidic platforms for multiplex detection [21]. Therefore, this review summarizes recent advances in various microfluidic platforms for multiplex detection of infectious diseases (Figure 1). In particular, immunosensors and nucleic acid sensors that are based on microfluidics for multiplex detection of pathogens are discussed. The current challenges, commercialization, and prospects are also proposed to improve the development of more efficient multiplex microfluidic platforms, especially in the rapid and accurate diagnosis of infectious diseases.

## 2. Multiplex Immunosensors on Microfluidic Platforms

The microfluidic immunosensor is a well-developed diagnostic tool for detecting analytes at low concentrations, which use antibodies as the biological recognition element to convert an antibody-antigen binding event into a measurable physical signal [24]. In the microfluidic multiplex immunosensor, a series of discriminatory biomarkers are simultaneously recognized by the antibodies with high specificity and sensitivity, generating signals in proportion to the antigen concentration in the samples [16,25,26,27,28,29,30,31,32,33,34,35]. For infectious disease diagnosis, multiplex immunosensors based on microfluidic platforms mainly use spatial multiplexing and barcode multiplexing strategies. According to the principle of fluid propulsion, they can be classified into capillary, pressure-driven, centrifugal, electrokinetic, and acoustic systems [36,37]. Among them, capillary force-driven microfluidic platforms are widely used because they do not require external energy to enable continuous fluid automation, with lateral flow immunoassay (LFIA) being the most typical representative [36].

LFIA has become one of the most successful analytical techniques because it meets the ASSURED (affordable, sensitive, specific, user-friendly, rapid and robust, equipment-free, and delivered) criteria of the WHO [38]. The rapidity, simplicity, relative cost-effectiveness, and the ability to be used by unskilled personnel have contributed to the widespread acceptance of LFIA [38,39]. In fact, the global lateral flow assay market was estimated to be approximately $5.98 billion in 2019 and is expected to reach $10.36 billion by 2027 [39]. Simultaneous detection of multiple analytes is mainly realized by arranging multiple test lines (TL) in a single strip, which enables discrimination of different targets by spatial resolution [39,40]. There are three typical signal readout strategies for LFIA platforms, including colorimetric signal, surface-enhanced Raman scattering (SERS) signal, and fluorescent signal, which are discussed below.

**Figure 2:**
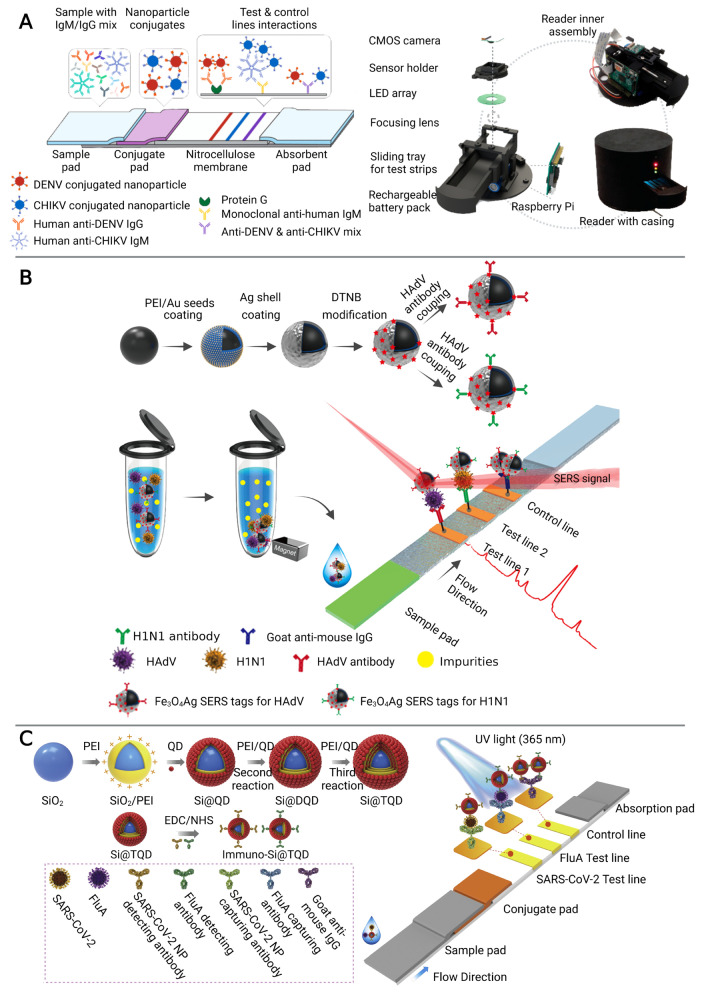
The multiplex detection of infectious diseases using microfluidic immunosensors. (**A**) Rapid diagnostic platform for DENV and CHIKV IgM/IgG antibodies, consisting of a multiplex color encoded lateral flow test strip and optical reader. Adapted with permission from Ref. [27]. Copyright 2019, American Chemical Society. (**B**) The multiplex detection of H1N1 and HAdV of the magnetic SERS-LFIA. Adapted with permission from Ref. [29]. Copyright 2019, American Chemical Society. (**C**) The multiplex detection of H1N1 and SARS-CoV-2 based on a dual-channel fluorescent immunochromatographic assay. Adapted with permission from Ref. [32]. Copyright 2021, Elsevier.

Colorimetric LFIA is the most commonly used for simple and rapid detection of pathogens [41,42]. The analytes (pathogens or their antigens) are first captured by antibodies on the strip, followed by the recognition of detection antibody which is usually conjugated with colorimetric readout elements such as gold nanoparticles (AuNPs) to produce a visible signal on the test line [42]. A number of LFIA platforms based on different nanoparticles have been developed for multiplex detection of infectious diseases [26,27,28]. For example, a LFIA platform using a color-mixing encoding and readout strategy was established (Figure 2A). After binding nanoparticles and IgM/IgG antibodies against dengue virus (DENV) and chikungunya virus (CHIKV), labeled antibody-nanoparticle complexes bind to the immobilized capture reagents on the different test lines in sandwich format to show different colors. By processing RGB data (from photographs on the LFIA) captured by the reader, the test results could be read semi-quantitatively. Furthermore, this platform has been validated in 50 human plasma samples, demonstrating that it can be used for infectious disease management by providing accessible, evidence-based laboratory diagnosis [27]. However, nanoparticle-based colorimetric assays have two inherent shortcomings: limited sensitivity and poor quantitative capability [43]. To improve the sensitivity and quantitative detection capability of LFIA in the diagnosis of infectious diseases, a SERS signal-based system [29,30,31] and a fluorescence signal-based strategy [32,33,44] have been recently reported. SERS-LFIA platforms integrate functional SERS-encoded nanoparticles (NPs), also known as SERS nanotags, into the LFIA system instead of the commonly used AuNPs as signal reporters [45,46]. This platform can provide specific (fingerprint feature), strong (high sensitivity), and stable (no photobleaching) SERS signals [46]. For example, Wang’s group developed a sensitive and quantitative SERS-LFIA platform using Fe_3_O_4_@Ag nanoparticles as magnetic SERS nanotags (Figure 2B), which could simultaneously detect human adenovirus (HAdV) and influenza A H1N1 viruses from human whole blood, serum, and sputum samples. The limits of detection (LOD) for HAdV and H1N1 were 10 and 50 pfu/mL respectively, which is 2000 times higher sensitivity than the standard colloidal gold strip method [29]. In general, fluorescent LFIA in which quantum dots (QDs) are considered as one of the most commonly used signal labels, show higher sensitivity than the conventional AuNPs-based LFIA due to their excellent luminescence properties [47]. A LFIA platform based on a dual-channel fluorescent immunochromatographic assay (ICA) was developed for ultrasensitive and simultaneous qualification of SARS-CoV-2 and influenza A virus. A high-performance quantum dot nanobead (QB) was fabricated by adsorbing multiple layers of dense quantum dots (QDs) on the SiO_2_ surface and used as a highly luminescent label of the ICA system to ensure the high-sensitivity and stability of the assay. The LOD was 5 pg/mL for SARS-CoV-2 antigen and 50 pfu/mL for H1N1 within 15 min, respectively. In addition, this platform showed high accuracy and specificity in throat swab samples with two orders of magnitude improvement in sensitivity compared to a conventional AuNP-based platform [32].

There are various commercial LFIA products. Such as, ActiveXpress (Edinburgh Genetics Ltd, Edinburgh, UK), Roche (SD Biosensor Inc./Roche Diagnostics, Basel, Switzerland), and Standard-Q (SD Biosensor Inc, Gyeonggi-do, Republic of Korea) [48]. Despite the extensive LFIAs research and the availability of several commercial products for detection, some challenges remain. First, the sensitivity should be enhanced by fabrication of high-resolution instrumentation and label materials with high response signal [33]. Second, the false positives may be reduced by multi-signal synchronous detection based on multiplexed type and multi-signal type [49]. Third, the false negatives caused by mutations are required to decrease through selecting new immune targets in a timely manner [50,51].

## 3. Multiplex Nucleic Acid Sensors on Microfluidic Platforms

Nucleic acid is another biomarker that can be used for the diagnosis of infectious diseases, due to its unique and outstanding characteristics (e.g., molecular recognition, biocompatibility, functionalization, and programmability), which endow nucleic acids with potential application as powerful sensing elements and provide key information of specific genes and species [52]. PCR is typically considered as the gold standard detection method of nucleic acid-based diagnosis, but involves costly/advanced equipment and skilled personnel, so it cannot be easily combined with microfluidic technology [53]. Therefore, isothermal amplification and clustered regularly interspaced short palindromic repeats (CRISPR) and CRISPR-associated proteins (CRISPR/Cas) system, which have the advantage of low-cost, reliability, and do not require for bulky equipment, can be compatible with the microfluidic platforms for nucleic acid detection [20,54]. Based on the spatial separation of detection sites on microfluidic platforms, the multiplex detection of different nucleic acids can be realized [14]. Multiplex nucleic acid sensors on microfluidic platforms mainly include polymer-based microfluidic chips, paper-based microfluidic devices, and droplet-based microfluidic devices [55]. They are characterized by cost-efficiency, portability, low sample consumption (µL-fL), miniaturization (with dimensions of tens to hundreds of micrometers chambers), simplicity (no training is required), and multiplex detection (providing multiple spatially separated detection channels) [56]. In this section, we will discuss nucleic acid sensors on microfluidic platforms for multiplex diagnosis of infectious diseases [57,58,59].

### 3.1. Polymer-Based Microfluidics

Fabrication of microfluidic devices is an important step in integrated automated nucleic acid sensing. In this regard, polymers (e.g., polydimethylsiloxane, PDMS) are one of the most common materials, due to their advantages such as cost-effectiveness, good biocompatibility, and simple fabrication protocol [20]. Polymer-based microfluidic chip is highly automated (multistep continuous reactions can be realized via sophisticated microstructures) and integrated, which has been widely used for infectious disease detections [60].

**Figure 3:**
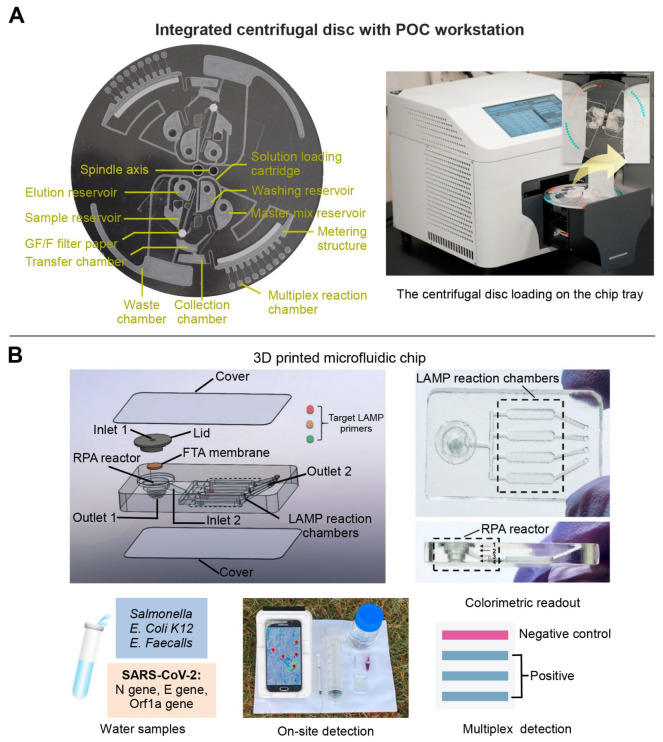
The multiplex detection of infectious diseases of polymer-based microfluidic chip. (**A**) A centrifugal microfluidic chip equipped with a glass-filter extraction column for purifying nucleic acid and multiple reaction chambers for multiplex detection. Adapted with permission from Ref. [63]. Copyright 2021, Elsevier. (**B**) A 3D printed microfluidic chip integrated on-chip nucleic acid extraction, two-stage isothermal amplification, and colorimetric detection. Adapted with permission from Ref. [70]. Copyright 2021, Elsevier.

Depending on the presence or absence of moving mechanical parts, fluid flow support techniques for polymer-based microfluidic chips are generally divided into mechanical drive (e.g., centrifugal force drive and micropump drive) and nonmechanical drive (e.g., electric drive and capillary force drive) [61,62]. The mechanical drive chips have the advantages of easy large-scale integration, high drive pressure, wide range of flow rates, and high adaptability. For example, Nguyen et al. designed a centrifugal disc equipped with a glass filter extraction column and multiple reaction chambers. Such a portable analyzer could simultaneously detect four pathogens of upper respiratory diseases within 90 min (Figure 3A) [63]. The non-mechanical drive is also suitable for integration, operation, and accurate control [61,63,64]. A pump-free microfluidic chip with capillary force drive was developed by Ciftci et al., which could directly collect the nucleic acid amplification products through the extraction solution without loading or washing procedures, enabling accurate diagnosis of the Ebola, Zika, and dengue viruses simultaneously [64].

The other key element for multiplex detection of infectious diseases is the design of microfluidic channels. The general processing technology of the polymer-based microfluidic channel includes the hot pressing, injection molding, photolithography, and laser etching [65]. For example, Huang et al. used laser cauterization to fabricate a basement layer consisting of two sides: side A contained microstructures for the recombinase polymerase amplification (RPA) reaction, and side B for the loop-mediated isothermal amplification (LAMP) reaction. Thus, the two-step of isothermal amplification could be completed on the chip to achieve a higher detection sensitivity (10 copies/μL) [66]. Similarly, Choi et al. developed a CRISPR/Cas strategy nucleic acid amplification-free on a polymer-based microfluidic chip. The activated CRISPR-Cas 12a in the presence of viral DNA was combined with a Raman-sensitive system consisting of ssDNA-immobilized Raman probe-functionalized AuNPs on the graphene oxide/triangle Au nanoflower array. Using this platform, simultaneous detection of hepatitis B virus (HBV), human papillomavirus 16 (HPV-16), and HPV-18 could be achieved with high sensitivity range from 1 aM to 100 pM without any amplification steps [67]. However, these methods require sophisticated instrumentation and high cost, and are difficult to achieve rapid prototyping [68]. To address this problem, three dimensional (3D) printing technology has been gradually adapted for the fabrication of polymer-based microfluidic chip manufacturing [69]. For example, our group used 3D printing technology to develop a sensitive, multiplex colorimetric detection (SMCD) method for the detection of pathogens in wastewater samples (Figure 3B). This SMCD method integrated nucleic acid extraction, RPA, LAMP, and colorimetric detection into microfluidic chips, and detected multi-gene targets of SARS-CoV-2 and multiple human enteric pathogens from the wastewater. The detection time was about 60 min, which was half of the time required for the qPCR method. Moreover, a smart, connected, and on-site detection was achieved with a reporting framework embedded in a smartphone-based detection platform, which exhibited the rapid spatiotemporal epidemiological data collection potential regarding the transmission and persistence of infectious diseases [70].

Biological manufacturers have developed several polymer-based microfluidic commercial devices [e.g., Revogene (Meridian Bioscience, Cincinnati, OH, USA) and GenPlex^®^ (BOHUI, Beijing, China)] for the diagnosis of infectious diseases [1,71,72,73]. Nevertheless, weakness of polymer materials includes thermal conductivity and low thermal resistance. The surface modification of polymers also needs to be further explored [74,75]. In addition, a practical solution for the processing and integration of the whole microfluidic system should be presented and optimized [74]. Finally, microfluidic devices need to be tested in large-scale clinical trials before being commercialized [75].

### 3.2. Paper-Based Microfluidics

The paper-based microfluidic device is a miniature laboratory analysis system that utilizes a paper substrate to replace the conventional substrates (e.g., quartz, silicon, and glass) [76]. There are several advantages of paper-based microfluidic devices: (1) paper is cheaper than conventional substrates; (2) the paper itself has a capillary effect, which can guide reagent flow without external forces; and (3) the flexibility and elasticity of paper facilitate customization [77,78]. Therefore, paper-based microfluidic devices, including lateral flow assays (LFA) and microfluidic paper-based analytical devices (μPAD) are widely applied to the multiplex detection of infectious diseases [79].

**Figure 4:**
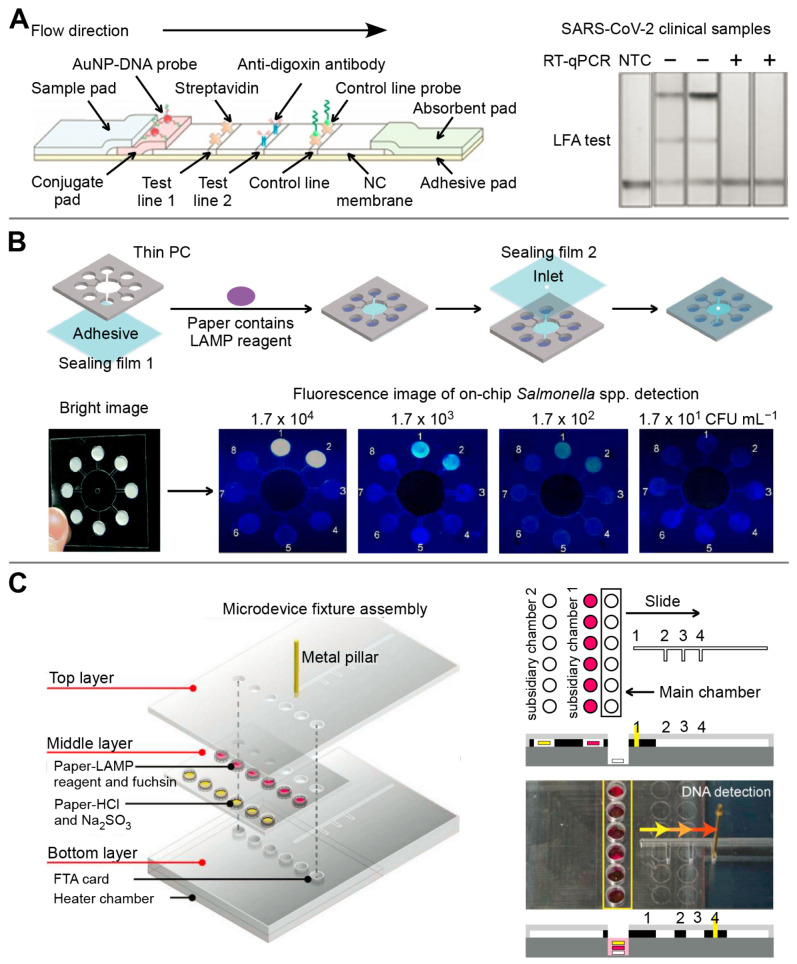
The multiplex detection of infectious diseases based on paper-based microfluidic devices. (**A**) The multiplex detection for E and ORF1ab genes of SARS-CoV-2 based on CRISPR/Cas9-mediated LFA. Adapted with permission from Ref. [58]. Copyright 2021, WILEY. (**B**) Fluorescent μPAD-based LAMP was prepared by simple craft-cutting for the simultaneous detection of four pathogens. Adapted with permission from Ref. [86]. Copyright 2018, Royal Society of Chemistry. (**C**) The colorimetric slidable μPAD was prepared by etching method for simultaneous detection of *Salmonella* spp., *Staphylococcus aureus,* and *Escherichia coli O157:H7*. Adapted with permission from Ref. [87]. Copyright 2019, Elsevier.

Generally, the LFA consists of a sample pad, a conjugate pad, a nitrocellulose filter membrane, an absorbent pad, and a back card. Each part has varied degrees of overlapping to ensure the continuity of sample flow [80]. Simultaneous detection of different targets can be achieved by fixing and modifying multiple identification elements and signal transduction elements on the LFA test strip [58]. Zhang’s group reported that SHERLOCKv2 can simultaneously detect dengue and Zika virus single-stranded RNA using a fantastic CRISPR/Cas detection system on the LFA platform [81,82]. Similarly, a CRISPR/Cas9-mediated triple-line LFA (TL-LFA) was combined with a multiplex RPA to achieve rapid and simultaneous detection of the E and ORF1ab genes of SARS-CoV-2 in a single strip test (Figure 4A). The TL-LFA showed high sensitivity with an LOD of four copies/mL. The total detection time, including sample pretreatment and analysis, was within 60 min [58].

In 2007, Whiteside’s group at Harvard University first proposed the concept of µPAD [83]. Generally, the fabrication technologies of μPADs are categorized as photolithography, PDMS stamping and printing, laser cutting, engraving, plasma treatment, screen printing, and vapor phase deposition [84]. The µPAD can simultaneously quantify multi-component objects, which is hardly achieved by conventional test papers [84,85]. Therefore, μPAD has gained significant interest as a promising analytical platform for point-of-care testing in the last ten years [85]. Nae Yoon Lee’s group developed a series of μPADs for the detection of several infectious diseases [86,87]. For example, a μPAD based on LAMP was fabricated to detect *Escherichia coli O157:H7*, *Salmonella* spp., *Staphylococcus aureus,* and *Cochlodinium polykrikoides*, followed by on-chip fluorescent readouts (Figure 4B). This platform can detect as low as 0.12 ng/μL and 0.13 ng/μL for *S. aureus* and *E. coli O157:H7* DNA, respectively [86]. Moreover, a slidable μPAD contained three layers to accomplish DNA extraction, LAMP reaction, and multiplex colorimetric signal output sequentially (Figure 4C). A novel colorimetric fuchsin-based method was used to detect LAMP amplicons. The limit of detection of *Salmonella* spp., *Staphylococcus aureus*, and *Escherichia coli O157:H7* were 3.0 × 10^1^, 3.0 × 10^2^, and 3.0 × 10^1^ CFU/sample, respectively [87].

There are several successful commercialized LFAs (e.g., urine dipstick and pregnancy test kit) [88,89] and μPADs (e.g., Diagnostics For All and INSiGHT) [90,91]. However, problems remain. Firstly, the LFAs often allows only qualitative/semi-quantitative results, and thus researchers have tried to solve this by introducing smart phone attachments to achieve more accurate result analysis [92]. In addition, the insufficient validation on their compatibility with real sample matrices may cause discrepancy between the detection results obtained from real samples and standard samples and can eventually require major modification of the device design [93]. Thus, the examination of μPADs using clinical samples in a real environment is deemed to be essential [90]. Finally, the integration degree of the µPADs can be improved by adding sample processing modules which can simplify the operational steps and improve the integrated capability of the devices [94].

### 3.3. Droplet-Based Microfluidics

A droplet-based microfluidic device is an alternative strategy for large-scale and parallel biological and chemical reactions [95]. The key technology is to generate small and mono-dispersed droplets (picoliter to nanoliter level) under high frequency (∼kHz) and precise control. Generally, microdroplets are generated from two incompatible liquids as a continuous phase and discrete phase, respectively, and different size distributions can be formed by controlling the microsphere structure and flow ratio of the two phases (the volume of microdroplets varies greatly from microliters to femtoliters) [96]. Meanwhile, the spatial separation of detection sites on the chips can be used as independent bioreactors to effectively distinguish different reactions [95,96]. Therefore, high-throughput droplet-based microfluidic devices enable the large-scale screening and multiplex detection of infectious diseases [97].

**Figure 5:**
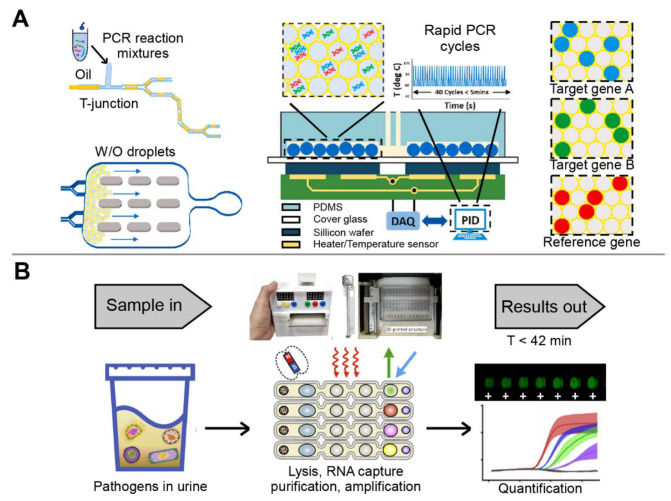
The multiplex detection of infectious diseases using droplet-based microfluidic devices. (**A**) Droplet PCR detection system fabricated by T-type structure method. Adapted with permission from Ref. [100]. Copyright 2021, Elsevier. (**B**) Automatic droplet-based microfluidic platform controlled by magnetic force. Adapted with permission from Ref. [59]. Copyright 2021, Elsevier.

Depending on the mode of droplet generation, droplet-based microfluidic devices can be divided into active and passive modes. The passive mode requires additional energy input to generate droplets, while the active mode generates droplets without external propulsion [98]. The passive modes, which do not require programmable syringe pumps or other automated instrumentation to control fluid flow, have been designed to construct droplet-based microfluidic devices [99]. For example, the first combination of droplet digital PCR technique was developed to simultaneously detect ORF1ab and N genes of SARS-CoV-2 (Figure 5A). Compared with the standard qPCR method, the droplet digital PCR system showed similar accuracy but with a lower turnaround time and a lower false-negative results [100]. In another study, a microdroplet platform integrating multiple LAMP, scorpion-shaped probes, and fluorescence microscopic counting, was developed using the flow-focusing method. The platform successfully detected Hepatitis C virus (HCV) and HIV from clinical plasma samples, with an LOD of four copies/reaction [101].

In contrast, active designs enable on-demand generation of droplets with a short response time and a better control of droplet size, content, and motion [102]. Specifically, compared with a few seconds or even minutes in a passive approach, the response time can be reduced to a few milliseconds in an active method. In addition, active methods control droplet size and production rate with higher flexibility and additional handles, and allow on-demand droplet generation, thus greatly promoting practical applications of microfluidic droplets [103]. However, in the passive approach, it is almost impossible to control droplet size and generation frequency independently because they are interrelated through mass conservation [99,102]. To date, the cutting-edge active techniques mainly apply magnetic, electrical, thermal, optical, mechanical, and centrifugal methods, by which magnetic, electrical, and centrifugal forces are introduced, and the viscosity, flow, interfacial tension, fluid density, and channel wettability are varied [104]. A cost-effective and automated multiplex micro-droplet detection platform was created using 3D-printed structural parts incorporated with off-the-shelf mechanic/electronic components (Figure 5B). The platform used magnetic force to control the linear displacement of the multichannel array chip, and seamlessly integrated multiple steps including bacterial lysis, RNA extraction, and amplification through droplet combination. The sample–answer assay could be completed within 42 min, with 100% concordance with qPCR [59]. In addition, the combinatorial arrayed reactions for multiplex evaluation of nucleic acids (CARMEN) were developed for scalable and multiplex pathogen detection. As a result, the platform simultaneously detected 169 human-associated viruses with at least 10 published genome sequences. In addition, SARS-CoV-2 was also detected by incorporating CARMEN-Cas 13a and an additional crRNA. The multiplex and throughput abilities of CARMEN made it practical to scale-up, as miniaturization reduced reagent cost per test by more than 300-fold [2]. Therefore, CARMEN enables large-scale CRISPR-based diagnosis, which is an important step toward routine, comprehensive infectious disease surveillance to improve patient care and public health [105,106].

Significant progress has been made in droplet-based microfluidics and some commercial products (e.g., 10× *g* and Drop-seq) have been developed [107,108]. To largely facilitate commercialization, several challenges remain to be addressed. For passive mode, it occurs at quite low flow rate ratio, tip-streaming is stable typically in less than few minutes, and then is destabilized by the variation in flow rate of syringe pumps [103]. Thus, it is indispensable to design a system that facilitates a stable tip-streaming over a long period of time [103,109]. In active design, the system should be parallelized and miniaturized. Furthermore, the piezoelectric dispenser and pulse laser-driven droplet generation show great potential to develop the fast-responding actuation and smart design of microfluidic junctions [108].

## 4. Conclusions and Future Prospects

Recent research on multiplex microfluidic detection platforms (Table 1) have demonstrated their potential for developing accurate, convenient, and rapid diagnoses of infectious diseases. LFIA is known as an ideal diagnostic assay characterized by fast, easy operation, durable stability, and low cost. Polymer-based microfluidic chips are a highly automated and integrated microfluidic platform. Paper-based microfluidic devices offer advantages such as ease of processing, control of reagent flow without external forces, and ease of customization. Droplet-based microfluidic devices are an alternative strategy for large-scale and parallel biological and chemical reactions, providing advantages of high throughput, low-cost, and multiplex detection. The industrialization of microfluidic platforms is also still in its infancy, and challenges such as liquid leakage and difficulties in reusability need to be improved by enhancing functional modularity of sample processing and target detection, as well as by automating platform fabrication and finding cost-effective substrate substitutes. Moreover, to improve the sensitivity and specificity of detection, the combination of two or more technologies may enhance the signal output or minimize interference [22]. Finally, cross-contamination and sensitivity attenuation of multiplex detections, and biosafety, data security, and privacy issues of microfluidic platforms should also be examined. A cohesive collaboration of industry and academic institutes will be increasingly desired to put microfluidic platforms into mass production and market distribution. In summary, microfluidic platforms have the outstanding advantages of integration, miniaturization, automation, and high-throughput, and the detection assays own the capabilities of portable signal readouts, simplicity, sensitivity, and specificity. Therefore, it is expected that microfluidic platforms integrated with various detection methods will provide a conceptually novel tool for infectious disease diagnoses.

## Figures and Tables

**Table 1:** Summary of multiplex microfluidic platforms based on sensor types.

Type of Sensors	Infectious Disease	Sample	LOD	Assay Time (min)	Ref
immunosensor	H1N1 and HAdV	whole blood, serum, and sputum	50 and 10 pfu/mL	30	[29]
	DENV and CHIKV	serum	100 ng/mL	30	[27]
	YFV, DENV, and ZEBOV	serum	150 ng/mL	-	[26]
	SARS-CoV-2 and H1N1	throat swab	5 pg/mL and 50 pfu/mL	15	[32]
	anti-SARS-CoV-2 IgM and IgG	serum	1 and 0.1 ng/mL	15	[30]
	SARS-CoV-2 spike and nucleocapsid protein antigens	saliva and nasal swab	0.5 pg/mL	35	[33]
nucleic acid sensor	FHV, MPF, BDB and CDF	oropharyngeal samples	1 × 10^4^, 1 × 10^3^, 1 × 10^3^, and 1 × 10^3^ copies/μL	90	[63]
	SARS-CoV-2 and measles	nasopharyngeal swab	10 copies/μL	60	[66]
	HBV and HPV (16.18)	plasmid	1 aM	20	[67]
	SARS-CoV-2 and human enteric pathogens	Waste-water	1 × 10^2^ GE/mL and 5 × 10^2^ CFU/mL	60	[70]
	gene E and ORF1ab gene of SARS-CoV-2	nasopharyngeal swab	4 copies/ml	60	[58]
	*Escherichia coli O157:H7, Salmonella* spp. and *Staphylococcus aureus*,	milk	0.13 ng/μL, 1.7 × 10^2^ CFU/mL and 0.12 ng/μL	70	[86]
	*Salmonella* spp., *Staphylococcus aureus,* and *Escherichia coli O157:H7*	juice and milk	3.0 × 10^1^, 3.0 × 10^2^ and 3.0 × 10^1^ CFU/sample	75	[87]
	ORF1ab and N genes of SARS-CoV-2	throat swab	5 and 10 copies/test	5	[100]
	HCV and HIV	plasma	4 copies/reaction	60	[101]
	*MP, CA-16 and EV-71*	urine	10^2^ copies/reaction	42	[59]

Notes: H1N1, influenza A; HAdV, human adenovirus; DENV, dengue virus; CHIKV, chikungunya virus; YFV, yellow fever virus; ZEBOV, Zaire Ebola virus; FHV, feline herpesvirus 1; MPF, *Mycoplasma felis*; BDB, *Bordetella bronchiseptica*; CDF, *Chlamydophila felis*; HBV, Hepatitis B virus; HPV 16 and 18, human papillomavirus 16 and 18; HCV, Hepatitis C virus; HIV, human immunodeficiency virus; *MP, Mycoplasma pneumoniae*; *CA-16*, *Coxsackievirus A type 16*; *EV-71, Enterovirus 71.*

## Data Availability

Not applicable.
